# The Impaired Subcortical Pathway From Superior Colliculus to the Amygdala in Boys With Autism Spectrum Disorder

**DOI:** 10.3389/fnint.2022.666439

**Published:** 2022-06-17

**Authors:** Yiting Huang, Mark Vangel, Helen Chen, Maya Eshel, Ming Cheng, Tao Lu, Jian Kong

**Affiliations:** ^1^School of Life Sciences, Beijing University of Chinese Medicine, Beijing, China; ^2^Department of Psychiatry, Massachusetts General Hospital and Harvard Medical School, Charlestown, MA, United States; ^3^Department of Medicine, Massachusetts General Hospital and Harvard Medical School, Charlestown, MA, United States

**Keywords:** autism spectrum disorder, fMRI, DTI, amygdala, SC, pulvinar

## Abstract

**Objective:**

Increasing evidence suggests that a subcortical pathway from the superior colliculus (SC) through the pulvinar to the amygdala plays a crucial role in mediating non-conscious processing in response to emotional visual stimuli. Given the atypical eye gaze and response patterns to visual affective stimuli in autism, we examined the functional and white matter structural difference of the pathway in boys with autism spectrum disorder (ASD) and typically developing (TD) boys.

**Methods:**

A total of 38 boys with ASD and 38 TD boys were included. We reconstructed the SC-pulvinar-amygdala pathway in boys with ASD and TD using tractography and analyzed tract-specific measurements to compare the white matter difference between the two groups. A region of interest-based functional analysis was also applied among the key nodes of the pathway to explore the functional connectivity network.

**Results:**

Diffusion tensor imaging analysis showed decreased fractional anisotropy (FA) in pathways for boys with ASD compared to TD. The FA change was significantly associated with the atypical communication pattern in boys with ASD. In addition, compared to TD, we found that the ASD group was associated with increased functional connectivity between the right pulvinar and the left SC.

**Conclusion:**

Our results indicated that the functional and white matter microstructure of the subcortical route to the amygdala might be altered in individuals with autism. This atypical structural change of the SC-pulvinar-amygdala pathway may be related to the abnormal communication patterns in boys with ASD.

## Introduction

Autism is a highly heterogeneous neurodevelopmental spectrum disorder, characterized by atypical social communication and restrictive and repetitive behaviors and interests (Lai et al., 2014). Among the triad of the symptoms, impairment or absence of social instinct is proposed to lie at the core of autism (Chevallier et al., 2012).

Face perception remains the main focus of autism research because it is a fundamental component of social recognition. Compared to typically developing (TD) individuals, people with autism spectrum disorder (ASD) were reported to have difficulties in recognizing the identity of a face (Morin et al., 2015; Shah et al., 2016) and decoding the emotional information from its expression (Fridenson-Hayo et al., 2016; Martínez et al., 2019). Though the underlying pathophysiology of the impaired face perception in autism remains unclear, there is a theory proposing that avoidance of eye contact might account for the pattern of face impairments in autism (Tanaka and Sung, 2016; Madipakkam et al., 2017).

Evidence from eye-tracking studies in individuals with ASD indicated that disorganized scanning patterns occur while viewing a static face. Compared to TD individuals, people with ASD concentrated less on the core features of the face (e.g., eye, nose, and mouth) and paid more attention to the external factors (e.g., hair and chin) (Wan et al., 2019). When the individuals with ASD did attend to the core features, they directed their gaze patterns to the mouth area and spent less time inspecting the eyes (Spezio et al., 2007).

Despite the importance of eyes in recognizing face identity and signaling emotions, they can be perceived as a potentially threatening area of the face for individuals with autism. A previous study showed that individuals with ASD had a stronger skin conductance reaction to direct eye gaze when compared with TD peers, indicating a hyperphysiological arousal (Kylliäinen et al., 2012). Indeed, people with ASD anecdotally described looking into others’ eyes as being an unpleasant, even painful experience (Robison, 2007). A previous neuroimaging study showed that individuals with ASD gazed less toward the eye region of the face compared to the TD group, and experienced elevated amygdala (AMG) activation when experimentally manipulating their gaze toward the eye region. Moreover, among individuals with ASD, a reduced eye gaze was associated with higher threat ratings for neutral faces (Tottenham et al., 2014).

Though the pathophysiology behind the atypical eye gaze pattern and its potential arousal of threatening emotions in individuals with ASD remain unclear, the processing of affective properties of stimuli is widely believed to stem from the AMG (Pessoa, 2017). Recent studies have prompted an increasing interest in a subcortical pathway, originating from the superior colliculus (SC) *via* the pulvinar (PUL) to the AMG, that mediates automatic attention to the face and eyes and the non-conscious perception of emotions (Kleinhans et al., 2011; Maior et al., 2012; McFadyen et al., 2020).

Previous neuroimaging studies have reported atypical responses in the SC, PUL, and AMG to faces and eye gaze in ASD (Kleinhans et al., 2011; Zürcher et al., 2013; Hadjikhani et al., 2017). For instance, by using functional magnetic resonance imaging (MRI) (fMRI), Hadjikhani et al. (2017) found an abnormally high activation in the SC, PUL, and AMG in individuals with ASD when constrained to looking into the eyes of dynamic faces expressing different emotions (neutral, happy, angry, and fearful) rather than when freely viewing them. Another fMRI study used mixed cues of fearful faces appearing to have averted or direct gazes. In this study, researchers found increased activation of the regions in the subcortical route (SC, PUL, and AMG) in individuals with ASD when presented with a direct fearful gaze but not an averted fearful gaze (Zürcher et al., 2013). Both of the studies indicate that the increased activation of this subcortical route in individuals with ASD might not necessarily be related to fearful face recognition if the gaze is constrained to the eyes. This may also explain the inconsistent results of another study examining the functional abnormalities in this subcortical route during supraliminal fearful face processing, where the results showed that individuals with ASD failed to engage those subcortical regions when compared with the engagement of the control groups (Kleinhans et al., 2011), as the study did not specify the eye gaze areas during the experiment.

To date, most of the studies examining the functional abnormalities of the regions in the SC-PUL-AMG route are task-based and have rarely investigated the structural integrity of the pathway. Diffusion tensor imaging (DTI) has the advantage of measuring the microstructural architecture of cellular membranes; larger average spacing between membrane layers increases the apparent diffusivity, whereas smaller spaces lead to lower apparent diffusivities. This sensitivity makes DTI a powerful method for detecting microscopic differences in tissue properties (Alexander et al., 2007). Fractional anisotropy (FA) is the most used parameter in tensor measurement. It is a summary of the microstructural change but does not specify the type of change (e.g., axial or radial). The mean diffusivity (MD) represents the inverse measures of the membrane density and is sensitive to cellularity, edema, and necrosis. The apparent diffusivities in the directions parallel and perpendicular to the WM tracts are the axial and radial diffusivities, AD and RD, respectively, which provide more direct measures of the microstructural dimensions (Alexander et al., 2011). A previous study, when testing the looming-evoked defensive response in individuals with ASD, reported a reduced FA of the SC-PUL-AMG pathway in the non-responding group when compared with the FA of the responding group (Hu et al., 2017). However, the structural integrity of the tract has yet to be compared in individuals with ASD and TD.

This study aimed to investigate both the functional and structural characteristics of the SC-PUL-AMG pathway associated with ASD. Specifically, we reconstructed the SC-PUL-AMG pathway in boys with ASD and TD by using probabilistic tractography and compared the four most commonly used diffusion measurements [FA, MD, AD, and RD] between the ASD and TD groups. In addition, we applied a region of interest (ROI)-based functional analysis among the bilateral SC, PUL, and AMG to explore the functional ROI connection network within the key nodes of the pathway. In the aforementioned studies concerning this subcortical pathway, Hadjikhani et al. (2017) had mostly boys in their study and Hu et al. (2017) only included male participants. We only included males for analysis to reduce the gender bias.

Given the atypical eye gaze pattern and abnormal response to the visual affective stimuli reported in individuals with ASD, we hypothesized that structural integrity and functional connections of the SC-PUL-AMG pathway might be impaired in children with ASD compared with their matched peers.

## Materials and Methods

### Subjects

Subjects were selected from two data sites: New York University (NYU, both sample 1 and sample 2) and Trinity Center for Health Sciences (TCD) from the Autism Brain Imaging Data Exchange II (ABIDE II^1^). Inclusion criteria were: (1) boys aged 6–19 years old; (2) full-scale IQ (FIQ) scores > 80; (3) diagnosis of ASD based on DSM-IV-TR and assessed with the Autism Diagnostic Interview-Revised (ADI-R), the Autism Diagnostic Observation Schedule (ADOS), or both; (4) resting-state fMRI, structural MRI, and DTI data were collected, and (5) all individuals completed the child behavior checklist (CBCL), a widely used caregiver report form identifying behavioral and emotional problems in children and adolescents (Achenbach and Rescorla, 2013), which includes anxiety/depressed effective measurement as a subscale. In addition, the Social Responsiveness Scale (SRS) (Constantino et al., 2003), a 65-item rating scale that quantifies the severity of social impairment within ASD, was also collected for all subjects. All procedures from these two sites were approved by their local Institutional Review Boards.

### Image Data Acquisition

Diffusion tensor imaging (DTI), resting-state fMRI scans, and anatomical scans were acquired on Siemens Allegra (Siemens Healthcare GmbH, Erlangen, Germany; NYU) and Philips 3T Achieva (Philips Healthcare, Best, Netherlands; TCD) MRI scanners. All the subjects were asked to relax with their eyes open in the scanner. Site-specific protocols are given in Supplementary Method 1.

### Region of Interests

We chose SC, PUL, and AMG as our ROIs. The masks of these ROIs were all created in standard MNI space using FSL. For the AMG binary mask, we used the probabilistic Harvard-Oxford Subcortical atlas at a threshold of at least 50% probability. For the PUL, we used the parcellated PUL mask generated by Barron et al. (2015), merged the five clusters together, and used FSL to manually fill any holes in the resultant binary mask. Finally, for the SC, we used the SC binary mask generated by McFadyen et al. (2019), who reconstructed the SC-PUL-AMG subcortical pathway in 622 participants. All the selected ROIs were visually inspected in MRIcron^2^ before analysis.

### Diffusion Tensor Imaging Data Analysis

The diffusion-weighted images were preprocessed using FMRIB Software Library, version 6.0.3 (Woolrich et al., 2009).^3^ First, we used eddy_openmp to correct for eddy current-induced distortions and participant movements (Andersson et al., 2016). The “replace outliers” option was used to identify outliers owing to participant motion and to replace the slices by using a non-parametric prediction based on GPR (Andersson and Sotiropoulos, 2016). A binary brain mask was extracted from the subjects’ T1-weighted structural images. Together with corrected diffusion-weighted data, the *b*-value and *b*-vector were used to fit the diffusion tensor model at each voxel by DTIFIT. To acquire the probabilistic fiber tracking between the SC and AMG, bedpostx (Jbabdi et al., 2012) was first used to generate a Bayesian estimate of the probability distribution of different directions at each voxel. The three predefined ROIs were linearly transformed into the native space of each subject. Then fiber tracking was performed using FSL’s probatrackx2 (Behrens et al., 2003, 2007), which generated streamlines connecting voxels from an originating seed ROI to voxels in a target ROI. The following settings were used for tractography: simple Euler streamlining, number of samples per voxel = 5,000, number of steps per samples = 2,000. Step length = 0.5 mm, loop check, curvature threshold = 0.2, subsidiary fiber volume fraction threshold = 0.01, and seed sphere sampling = 0. In addition, we assessed the head motion of diffusion MRI (dMRI) data by calculating the relative volume-to-volume framewise displacement (FD) (Roalf et al., 2016) as commonly used in fMRI studies.

Similar to previous studies (Rafal et al., 2015; Hu et al., 2017), to acquire a robust streamline, we performed fiber tracking in both directions, from the SC (seed ROI) *via* the PUL and AMG as a waypoint to the AMG (target ROI), and from the AMG (seed ROI) *via* the PUL and SC as a waypoint to the SC (target ROI) in both hemispheres of each subject. An additional coronal exclusion mask around the medial diencephalon was also placed to prevent the tract’s diversion into the stria terminalis tract. The two-directional tracts threshold at 10% was binarized and normalized to the standard space for each subject. Then the tracts were summed across subjects and averaged to produce final tract pathways as a group-level probability map in each hemisphere. These group probabilistic maps were set at a threshold that allowed the paths present in at least 50% of the subjects to be displayed. After manually inspecting the tractography generated from each subject, we found that the aforementioned DTI tractography model and parameters failed to track the anatomically appropriate (missed or misaligned) SC-PUL-AMG streamline from the subjects selected from NYU datasets due to the low resolution/quality of the dMRI data (NYU’s data acquisition voxel size is 3 mm × 3 mm × 3 mm, TR = 5,200 ms, 50 slices and the TCD’s data acquisition voxel size is 2 mm × 2 mm × 2 mm, TR = 20,244 ms, 65 slices). Thus, for DTI analysis, we only included subjects from the TCD data site.

To extract the FA value of the SC-PUL-AMG tract, we also ran part of the Tract-Based Spatial Statistics (TBSS) to generate an MN152 space-aligned group FA map. We first ran a non-linear registration to align all the FA images of each subject generated from DTIFIT to a 1 mm × 1 mm × 1 mm standard space. The FMRIB58_FA standard-space image was used as the default target image. The standard-space version of each subject’s FA image was then merged into a single 4D image file. The measures of white matter microstructure were extracted from the merged 4D FA map by applying the generated group-level SC-PUL-AMG tract. We also extracted the MD, AD, and RD using the same method described above. In addition, using the aforementioned method, we also extracted the FA, MD, AD, and RD for each subject from two data sites (NYU and TCD) by using the SC-PUL-AMG pathway obtained from the TCD data site as a template for exploratory purposes. Detailed results of the DTI analysis from the two data sites were presented in Supplementary Result 1.

The acquired FA, MD, AD, and RD were imported in RStudio version 3.5.2^4^ for further statistical analysis. Mixed design ANOVA was performed with a group (ASD vs. TD) as between-group factors, and hemisphere (left vs. right) as within-group factors to examine differences in FA, MD, AD, and RD. Age, FIQ, and CBCL anxiety scores were entered as covariates.

### Region of Interest-Based Functional MRI Analysis

The images were preprocessed in CONN version 18b^5^ (Whitfield-Gabrieli and Nieto-Castanon, 2012) and SPM 12^6^ using CONN’s default preprocessing pipeline. The preprocessing steps included slice-timing correction, realignment, normalization (3 × 3 × 3 mm^3^ in MNI space), and smoothing (6 mm). During preprocessing, the Artifact Detection Tool^7^ was used to detect outliers (>3 SD and/or >0.5 mm). The outliers were used for the subsequent scrubbing regression. The structural images were segmented and used to create gray matter, white matter (WM), and cerebral spinal fluid (CSF) masks of each subject. Then, linear regression using WM and CSF signals (CompCor; 5 components for WM and CSF), linear trend, subject motion (6 rotation/translation motion parameters and 6 first-order temporal derivatives), and outlier scrubbing was conducted to remove confounding effects. Afterward, the residual BOLD time series were band-pass filtered (0.008–0.09 Hz). To minimize the effects of head motion, subjects with mean FD (Power et al., 2012) exceeding 0.5 mm were excluded for functional analysis.

The ROI-based functional analysis was conducted by selecting the predefined six ROIs (left and right SC, PUL thalamus, and AMG, respectively) to create a 6 × 6 functional connectivity map. A bivariate correlation was used to determine total linear temporal associations between each of the resulting 15 ROI-based functional connections (omit the connection from an ROI to itself and only test for one direction). Second-level analysis of group differences in functional connectivity between ASD and TD was generated with a threshold value of 0.05, FDR-corrected at the seed level. Age, FIQ, center, and CBCL anxiety scores were included as covariates.

## Results

### Demographic and Clinical Characteristics

Demographic and clinical variables are given in Table 1. In total, 76 subjects were included in the study. A total of 38 boys with ASD (mean age, 10.8 years; age range, 5.6–19.3 years) and 38 boys with TD (mean age, 11.9, age range, 5.9–19.3 years) were selected based on the inclusion criteria. For ASD assessment, 38 subjects received ADI-R measurement. And 27 subjects were assessed with ADOS (ADOS-G). There were no significant differences between the ASD and TD groups in age and full-scale IQ (*P* > 0.05). There were significant differences in the CBCL anxiety score (*p* < 0.001) and SRS total score (*p* < 0.001) between the two groups. Age was included in all the group analyses as covariates to minimize the developmental effects.

**TABLE 1:** Participant demographics and clinical characteristics.

Characteristics	TCD		NYU		ASD vs. TD	
			
	ASD (*n* = 17)	TD (*n* = 17)	ASD (*n* = 21)	TD (*n* = 21)	*T*/*X*^2^	*p*
Age (years)	14.5 ± 3.3 (10–19)	15.0 ± 2.9 (10–19)	7.9 ± 1.4 (6–12)	9.5 ± 1.8 (6–12)	−1.2	*0.25*
Full-scale IQ	112.9 ± 12.6 (83–139)	118.6 ± 14.4 (81–142)	117.0 ± 14.6 (98–143)	115.1 ± 15.3 (92–144)	−0.4	*0.69*
**ADI-R**						
*Social*	16.8 ± 3.7 (11–24)	–	19.4 ± 5.8 (5–28)	–	–	–
*Communication*						
*Verbal*	13.6 ± 3.3 (8–19)	–	17.0 ± 5.0 (9–24)	–	–	–
*Non-verbal*	7.2 ± 3.0 (4–13)	–	10.0 ± 3.2 (3–14)	–	–	–
*RRB*	5.1 ± 2.8 (1–12)	–	5.9 ± 2.3 (2–10)	–	–	*-*
ADOS total score^†^	8.6 ± 2.5 (7–16)	–	11.9 ± 5.1 (4–24)	–	–	–
SRS total score	78.5 ± 9.0 (65–90)	43.8 ± 7.7 (35–58)	79.6 ± 14.6 (51–107)	45.6 ± 6.6 (34–59)	15.1	<*0.001*
CBCL anxiety score	62.4 ± 10.0 (50–79)	52.9 ± 4.8 (50–65)	61.3 ± 7.7 (50–74)	53.2 ± 5.0 (50–67)	5.4	<*0.001*

*Numbers are means ± standard deviation. Ranges are in bracket. NYU, New York University; TCD, Trinity Center for Health Science; ADI-R, autism diagnostic interview-revised; RRB, restrictive and repetitive behavior; ADOS, autism diagnostic observation schedule; SRS, social responsiveness scale; CBCL, child behavior checklist.*

*^†^ADOS-2 were used in NYU center, ADOS-G were used in TCD center.*

Previous studies had demonstrated that FIQ may be associated with white matter integrity (Chiang et al., 2009), and the PUL-AMG connectivity was shown to relate with anxiety levels in certain people (Hakamata et al., 2016; McFadyen, 2019). Therefore, we also included FIQ and CBCL anxious scores as covariates to minimize the potential confounding effects.

### Diffusion Tractography Analysis

Connections between the SC and AMG through the PUL were demonstrated in both hemispheres of 34 boys (17 boys with TD; 17 boys with ASD) from the TCD data site. The construction of the tract in the NYU data site could not be directly acquired by using the previously defined probabilistic tractography parameters due to the low resolution/quality of the dMRI data (see Section “Diffusion Tensor Imaging Data Analysis” for details). We thus used data from the TCD center for primary diffusion tractography analysis, and combined data from both TCD and NYU centers for exploratory diffusion tractography analysis.

**FIGURE 1:**
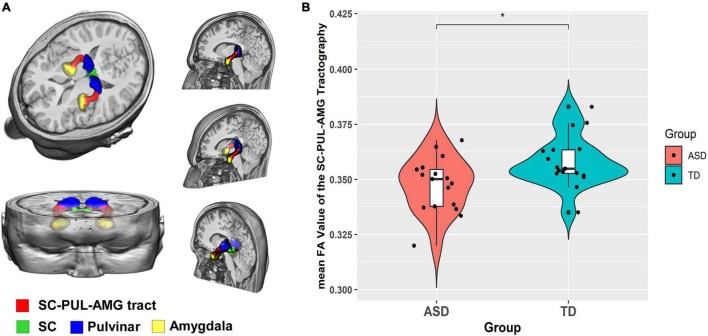
Diffusion tensor imaging probabilistic tracking result of SC-PUL-AMG concerning ASD symptoms. **(A)** Tractography reconstruction of the SC-pulvinar-amygdala tract in 3D view; **(B)** Violin plot of the distribution of the FA value of the tract in the ASD and TD group.

The group-level reconstruction of this streamline is shown in Figure 1A. Consistent with the previous studies (Rafal et al., 2015; Hu et al., 2017; McFadyen et al., 2019), the pathway ascends dorsally from the SC to the PUL and then proceeds rostrally above the temporal horn. After crossing from medial to lateral, the pathway terminates at the lateral AMG.

Between-group differences were statistically significant for FA [*F*(1,29) = 7.592; *p* = 0.010] (Figure 1B). The main effect of group remained statistically significant in FA value when we removed FIQ and CBCL anxiety score as covariates [*F*(1,29) = 6.853, *p* = 0.014]. ASD group (0.347 ± 0.013) had lower mean FA value compared to the TD group (0.358 ± 0.012). The results also yielded a significant main effect of hemisphere [*F*(1,32) = 14.73; *p* < 0.001]. There are no statistically significant interaction effects for FA. Statistically significant main effects of hemisphere were also found in MD [*F*(1,32) = 39.348; *p* < 0.001], AD [*F*(1,32) = 39.684; *p* < 0.001], and RD [*F*(1,32) = 37.306; *p* < 0.001]. No other significant between-group and interaction effects were found for MD, AD, and RD (Table 2).

**TABLE 2:** Tract-specific measurements of SC-pulvinar-amygdala in TCD data site.

	ASD, *n* = 17		TD, *n* = 17		Effect of group	Effect of interaction
		
	Left hemisphere	Right hemisphere	Left hemisphere	Right hemisphere		
	Mean ± SD	Mean ± SD	Mean ± SD	Mean ± SD		
FA	0.350 ± 0.013	0.345 ± 0.012	0.361 ± 0.011	0.356 ± 0.013	*F*(1,29) = 7.592, *p* = 0.010	*F*(1,32) = 0, *p* = 0.997
MD	(0.83 ± 0.02) × 10^–3^	(0.87 ± 0.03) × 10^–3^	(0.84 ± 0.02) × 10^–3^	(0.86 ± 0.03) × 10^–3^	*F*(1,29) = 0.001, *p* = 0.974	*F*(1,32) = 2.227, *p* = 0.145
AD	(1.13 ± 0.03) × 10^–3^	(1.18 ± 0.04) × 10^–3^	(1.15 ± 0.03) × 10^–3^	(1.18 ± 0.04) × 10^–3^	*F*(1,29) = 0.544, *p* = 0.467	*F*(1,32) = 2.231, *p* = 0.145
RD	(0.67 ± 0.02) × 10^–3^	(0.71 ± 0.03) × 10^–3^	(0.68 ± 0.02) × 10^–3^	(0.70 ± 0.03) × 10^–3^	*F*(1,29) = 0.261, *p* = 0.613	*F*(1,32) = 2.076, *p* = 0.159

*FA, fractional anisotropy; MD, mean diffusivity; AD, axial diffusivity; RD, radial diffusivity.*

As an exploratory analysis, we also applied the constructed tracts to extract FA values from the combined datasets (including DTI data from both NYU and TCD centers). We found a similar result of significant between-group differences for FA [*F*(1,71) = 11.973; *p* < 0.001] and a significant main effect of hemisphere [*F*(1,74) = 21.416; *p* < 0.001]. The tract-specific measurements are given in Supplementary Table 1 and results were detailed in Supplementary Result 1.

**FIGURE 2:**
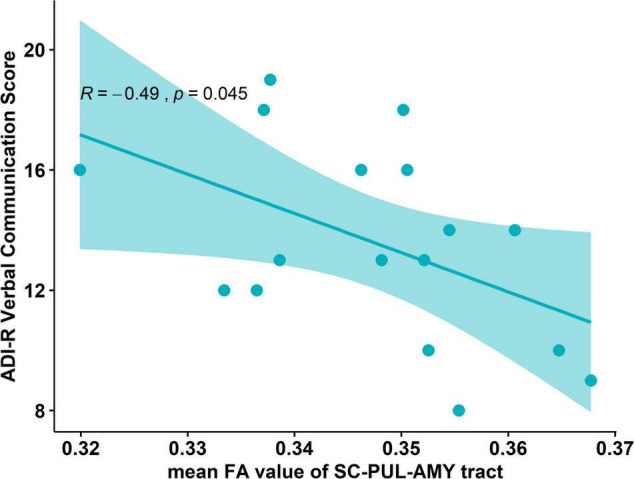
Correlation plot between the mean FA value and the behavior scores in the ASD group. Negative correlation between the mean FA value of the tract and the ADI-R verbal communication score (*R* = –0.49, *p* = 0.045).

We subsequently explored whether the anatomical differences in the FA of the tract were associated with the behavioral measurements in boys with ASD. Pearson’s correlation was calculated between tract-specific measurements and measures for autism symptoms, namely, ADI-R social score, communication score (including verbal and non-verbal), repetitive and restrictive behaviors, ADOS, and SRS total score. A statistically significant negative correlation was found between the mean FA value of the tract and the ADI-R verbal communication score (*R* = −0.49, *p* = 0.045, uncorrected, Figure 2). Other autistic trait-related measurements, namely, ADI-R social score (*R* = −0.05, *p* = 0.84), non-verbal communication (*R* = −0.24, *p* = 0.34), repetitive behaviors (*R* = −0.27, *p* = 0.28), ADOS total score (*R* = −0.11, *p* = 0.66), and SRS total score (*R* = 0.32, *p* = 0.21) were not significantly correlated with the FA value of the pathway in the boys with ASD.

Given the wide age range of the ASD group, partial correlation including age was also applied to examine the association between the FA value of the tract and the ADI-R verbal communication score. The significant level became less robust (*p* = 0.068).

### Region of Interest-Based Functional Analysis

One subject was excluded due to excessive head motion (mean FD exceeding 0.5 mm) during resting-state fMRI scan and there is no significant difference between ASD (0.26 ± 0.21) and TD (0.22 ± 0.17) group in head motion (*p* = 0.315). Therefore 75 subjects (37 ASD vs. 38 TD) were included in the functional connectivity analysis. The result of ROI-based functional analysis showed significantly increased functional connectivity between left SC and right PUL thalamus after FDR correction in boys with ASD. There was no significantly decreased connectivity between ROIs in boys with ASD.

To explore the association between the increased functional connectivity and the autism symptoms in boys with ASD, we extracted the Fisher’s *z* transformed values within 6 × 6 ROIs connectivity map and calculated the Pearson’s correlation between the significantly increased ROI-based functional connectivity and the behavioral measurements. There were no significant correlations between functional connectivity differences and the core trait of autism in boys with ASD.

Meanwhile, to investigate the potential associations between the white matter change and the functional connectivity change of the SC-PUL-AMY tract, we also examined the correlations between the FA values and Fisher’s *z* transformed values. There is no significant correlation found between these two measurements.

## Discussion

In this study, we investigated the white matter microstructure and functional connectivity of a subcortical pathway originating from the SC through the PUL to the AMG in boys with ASD. The results of the DTI analysis showed that compared to TD boys, boys with ASD have a lower FA value of the SC-PUL-AMG tract. Moreover, the reduced FA value was negatively associated with communication impairments in boys with ASD. We also found that boys with ASD are associated with increased functional connectivity between the right PUL and the left SC compared to the TD group.

This study reconstructed the SC-PUL-AMG pathway in boys with ASD and TD. The result confirmed the existence of the anatomical structure of this subcortical pathway as suggested by previous studies (Rafal et al., 2015; McFadyen et al., 2019). In boys with ASD, the decreased FA value of the SC-PUL-AMG tract was significantly associated with impaired communication ability. Though the interpretation of the DTI anisotropy measurements required cautions, FA values are usually sensitive to microstructural integrity (Alexander et al., 2007). Decreased FA may reflect the demyelination or axonal degeneration of the tract. Our results are consistent with a previous animal study that reported the synaptic connection of the SC-PUL-AMG pathway was impaired in the valproic acid mouse model of autism (Hu et al., 2017).

Within the nodes of the pathway, the SC is a critical structure in the midbrain that selects incoming visual stimuli based on salience and relevance to orienting behaviors, particularly shifts in gaze and attention, and pupil dilation as part of the orienting reflex (Gandhi and Katnani, 2011; Wang et al., 2012). By connecting with the AMG *via* the PUL, the pathway was thought to mediate the response to affective visual stimuli, a key component of social communication. Parents of autistic children frequently reported very early deviance in the development of basic non-verbal interpersonal skills, such as eye contact and facial expression (Dawson et al., 2000; Jones and Klin, 2013; Becerra-Culqui et al., 2018). Numerous studies have also noted deficiencies in the development of joint attention in autism, a complex communication form for the earlier capacity to share affect (Robertson et al., 1999). As social communication demands significant coordination of visual and emotional processing (i.e., eye contact and facial expressions), our results suggest that the impairments of this subcortical pathway might underlie the pathophysiology of the communication deficits in autism.

We also found distinct functional connectivity within the key nodes of the pathway compared to the structural connectivity. The results showed an increased resting-state functional connectivity between right PUL and left SC in boys with ASD. The variance between the structural and functional connectivity within the key nodes of the pathway remained unclear. As mentioned before, individuals with ASD showed signs of unconscious avoidance in eye contact (Akechi et al., 2014; Madipakkam et al., 2017). Moreover, a reduced eye gaze was associated with higher threat ratings for neutral faces (Tottenham et al., 2014). Studies also reported that individuals with ASD showed activation in the SC, PUL, and AMG in response to direct eye gaze (Robison, 2007; Zürcher et al., 2013). This suggests that the neural circuits that mediate the unconscious visual response might be directly linked with the function of the subcortical SC-PUL-AMG pathway. Furthermore, the pathologically hyperactivated resting-state connectivity between the core regions of the pathway may explain the exaggerated response toward direct eye gaze in individuals with ASD, which results in avoidance.

Furthermore, we have extracted the FA value of the tract based on the skeletonized mean FA generated from the TBSS approach, in which the intersubject variability has been considered (Smith et al., 2006). We did not normalize over the total white matter volume or white matter volume of a different major tract. Future studies may also consider these methods.

There are several limitations to this study. First, the tractography analysis is based on a relatively small dataset. Future studies incorporating a large sample size are desired. Moreover, the ASD population illustrates high comorbidity with anxiety disorder. With the potential relationship between the structural abnormalities of the pathway and anxiety disorder, the structural difference of the tract in boys with ASD should be interpreted with caution. A future anxiety level-matched ASD group could be further added to the analysis. Also, due to striking male prevalence in ASD, we only included males in this study. This was done to reduce heterogeneity, as the etiology of the sex bias in ASD diagnosis remains unclear (Werling and Geschwind, 2013). Future studies are needed to investigate the anatomical and functional connectivity alternations associated with both males and females and the gender differences. Finally, this is a cross-sectional study, therefore we cannot determine if the structural and functional alternation of the brain is caused by ASD. Further prospective studies are needed to validate our findings.

In summary, our results indicate that the functional and white matter microstructure of the subcortical route to the AMG might be altered in individuals with autism. The atypical structural change is associated with communication deficits in boys with ASD. Our findings may provide new insight into the atypical communication patterns associated with ASD.

## Data Availability Statement

The original contributions presented in this study are included in the article/Supplementary Material, further inquiries can be directed to the corresponding authors.

## Ethics Statement

The studies involving human participants were reviewed and approved by New York University School of Medicine; Irish Health Services Executive Linn Dara Child and Adolescent Research Ethics Committee; St. James’s Hospital/The Adelaide and Meath National Children’s Hospital. The patients/participants provided their written informed consent to participate in this study.

## Author Contributions

YH, JK, and TL: study design and conceptualization. YH, MC, HC, and ME: data downloading and trimming. YH, MV, and JK: data analysis and interpretation. YH, JK, HC, and ME: manuscript preparation. All authors contributed to the article and approved the submitted version.

## Conflict of Interest

The authors declare that the research was conducted in the absence of any commercial or financial relationships that could be construed as a potential conflict of interest.

## Publisher’s Note

All claims expressed in this article are solely those of the authors and do not necessarily represent those of their affiliated organizations, or those of the publisher, the editors and the reviewers. Any product that may be evaluated in this article, or claim that may be made by its manufacturer, is not guaranteed or endorsed by the publisher.

## Footnotes

1
http://fcon_1000.projects.nitrc.org/indi/abide/abide_II.html

2
https://www.nitrc.org/projects/mricron

3
https://fsl.fmrib.ox.ac.uk/fsl/fslwiki

4
https://rstudio.com/

5
https://sites.google.com/view/conn/

6
http://www.fil.ion.ucl.ac.uk/spm/

7
https://www.nitrc.org/projects/artifact_detect/
