# Supplementary material for: The Impaired Subcortical Pathway From Superior Colliculus to the Amygdala in Boys With Autism Spectrum Disorder
Source: Front Integr Neurosci. 2022 Jun 17;16:666439. doi: 10.3389/fnint.2022.666439 (PMC9247550; doi:10.3389/fnint.2022.666439)
Supplement: Supplementary file 1 [file Data_Sheet_1.docx]

**Supplementary materials:**

**Supplementary Method 1.** Magnetic resonance image acquisition parameters used in the current study.

All neuroimaging data from human participants were collected using a 3T Scanner (NYU: Siemens Allergra; TCD: Philips Achieva).

In the NYU data site (n= 42), structural MR imaging of the brain was performed with an axial 3D Magnetization Prepared Rapid Acquisition Gradient-Echo (MPRAGE) T1-weighted sequence (TR = 2,530 milliseconds; TE = 3.25 milliseconds; TI = 1,100 milliseconds; flip angle = 7°) with a 256 mm field of view (FOV), and 128 1.33 mm contiguous partitions at a 256×192 matrix. Diffusion-Weighted Echo-Planar (DW-EPI) images were acquired using a twice-refocused diffusion-weighted echo-planar image sequence with parameters TR = 5,200 milliseconds; TE = 78 milliseconds; 50 slices; acquisition matrix 64 × 64; field of view = 192 mm; acquisition voxel size = 3 × 3 × 3 mm; 64 non-collinear diffusion directions, uniformly distributed around a unit sphere with a b-value of 1000 s/mm^2^; one image with no diffusion weighting. The functional scans were collected using a customized multi-echo Echo Planar Imaging (EPI) sequence (TR = 2,000 ms; TE = 33 ms; flip angle = 90°, 33 slices, matrix = 64 × 64; FOV = 240 × 192 mm; acquisition voxel size = 3 × 3 × 4 mm; number of volumes = 197).

In the TCD data site (n=34), structural MR imaging of the brain was performed with an axial 3D Magnetization Prepared Rapid Acquisition Gradient-Echo (MPRAGE) T1-weighted sequence (TR = 8.4 milliseconds; TE = 3.9 milliseconds; TI = 1,150 milliseconds; flip angle = 8°) with a 230 mm field of view (FOV), and 190 0.9 mm contiguous partitions at a 256×256 matrix. Diffusion-Weighted Echo-Planar (DW-EPI) images were sequence was a single-shot spin echo EPI sequence acquired with parameters: TR = 20,244 milliseconds; TE = 79 milliseconds; 65 slices; acquisition matrix 124 × 124; field of view = 248 mm; acquisition voxel size = 2 × 2 × 2 mm; 61 non-collinear diffusion directions, uniformly distributed around a unit sphere with a b-value of 1500 s/mm2; one image with no diffusion weighting. The functional scans were collected using a customized multi-echo Echo Planar Imaging (EPI) sequence (TR = 2,000 ms; TE = 27 ms; flip angle = 90°, 37 slices, matrix = 80 × 80; FOV = 240 × 240 mm; acquisition voxel size = 3.5 × 3.5 × 3.5 mm; number of volumes = 37).

**Supplementary result 1.** Results of DTI analysis including imaging data from both NYU and TCD data sites

2 subjects were excluded due to excessive head motion (FD>1mm). In total, 36 ASD subjects and 38 TD subjects were included in the analysis. For group comparisons between ASD and TD in two combined data site, statistical analysis showed that between-group differences were statistically significant for the fractional anisotropy (FA) [F (1,69) = 10.379; *p* =0.002 ]. The results also yielded a significant main effect of hemisphere [F (1,72) = 21.108; *p* < 0.001]. There are no statistically significant interaction effects and other main effects for FA. Similar to the results of the dataset from TCD, statistically significant main effects of hemisphere were also found in MD [F (1,72) = 88.784; *p* < 0.001], AD [F (1,72) = 80.384; *p* < 0.001], and RD [F (1,72) = 87.425; *p* < 0.001]. No other significant between-group and interaction effects were found for MD, AD, and RD. The tract-specific measurements were presented in Table S1.

**Table S1** Fiber measurements of the SC-pulvinar-amygdala tract from the two combined data sites

|  | **ASD, n=36** | | **TD, n=38** | | Effect of group | Effect of interaction |
| --- | --- | --- | --- | --- | --- | --- |
|  | Left hemisphere  Mean ± SD | Right hemisphere  Mean ± SD | Left hemisphere  Mean ± SD | Right hemisphere  Mean ± SD |  |  |
| **FA** | 0.345 ± 0.016 | 0.340 ± 0.014 | 0.354 ± 0.011 | 0.350 ± 0.015 | F (1,69) =10.379  *p* = 0.002 | F (1,72) =0.042,  *p* = 0.838 |
| **MD** | (0.92 ± 0.09) ×10 ^-3^ | (0.96 ± 0.10) ×10 ^-3^ | (0.901 ± 0.08) ×10 ^-3^ | (0.94 ± 0.09) ×10 ^-3^ | F (1,69) =0.048,  *p* = 0.828 | F (1,72) =1.846,  *p* = 0.178 |
| **AD** | (1.25 ± 0.11) ×10 ^-3^ | (1.30 ± 0.12) × 10 ^-3^ | (1.25 ± 0.10) ×10 ^-3^ | (1.28 ± 0.11) ×10 ^-3^ | F (1,69) =0.089,  *p* = 0.767 | F (1,72) =1.574,  *p* = 0.214 |
| **RD** | (0.76 ± 0.08) ×10 ^-3^ | (0.74 ± 0.07) ×10 ^-3^ | (0.80 ± 0.09) ×10 ^-3^ | (0.77 ± 0.08) ×10 ^-3^ | F (1,69) =0.313,  *p* = 0.578 | F (1,72) =1.924,  *p* = 0.17 |
